# Genetic and Epigenetic Mechanisms Deregulate the CRL2^pVHL^ Complex in Hepatocellular Carcinoma

**DOI:** 10.3389/fgene.2022.910221

**Published:** 2022-05-18

**Authors:** Brenda C. Minatel, David E. Cohn, Michelle E. Pewarchuk, Mateus C. Barros-Filho, Adam P. Sage, Greg L. Stewart, Erin A. Marshall, Nikita Telkar, Victor D. Martinez, Patricia P. Reis, Wendy P. Robinson, Wan L. Lam

**Affiliations:** ^1^ Department of Integrative Oncology, British Columbia Cancer Research Institute, Vancouver, BC, Canada; ^2^ Department of Oncology, Hospital Sírio-Libanes, São Paulo, Brazil; ^3^ British Columbia Children’s Hospital Research Institute, Vancouver, BC, Canada; ^4^ Department of Medical Genetics, University of British Columbia, Vancouver, BC, Canada; ^5^ Department of Pathology and Laboratory Medicine, IWK Health Centre, Halifax, NS, Canada; ^6^ Department of Pathology, Faculty of Medicine, Dalhousie University, Halifax, NS, Canada; ^7^ Beatrice Hunter Cancer Research Institute, Halifax, NS, Canada; ^8^ Department of Surgery and Orthopedics and Experimental Research Unity (UNIPEX), Faculty of Medicine, São Paulo State University (UNESP), Botucatu, Brazil

**Keywords:** liver cancer, ubiquitin-proteasome, novel microRNAs, DNA copy number, DNA hypomethylation

## Abstract

Dysregulation of ubiquitin-proteasome pathway genes through copy number alteration, promoter hypomethylation, and miRNA deregulation is involved in cancer development and progression. Further characterizing alterations in these genes may uncover novel drug targets across a range of diseases in which druggable alterations are uncommon, including hepatocellular carcinoma (HCC). We analyzed 377 HCC and 59 adjacent non-malignant liver tissue samples, focusing on alterations to component genes of the widely studied CRL2^pVHL^ E3 ubiquitin ligase complex. mRNA upregulation of the component genes was common, and was correlated with DNA hypomethylation and copy number increase, but many tumours displayed overexpression that was not explained by either mechanism. Interestingly, we found 66 miRNAs, including 39 previously unannotated miRNAs, that were downregulated in HCC and predicted to target one or more CRL2^pVHL^ components. Several miRNAs, including hsa-miR-101-3p and hsa-miR-139-5p, were negatively correlated with multiple component genes, suggesting that miRNA deregulation may contribute to CRL2^pVHL^ overexpression. Combining miRNA and mRNA expression, DNA copy number, and methylation status into one multidimensional survival analysis, we found a significant association between greater numbers of alterations and poorer overall survival for multiple component genes. While the intricacies of CRL2^pVHL^ complex gene regulation require additional research, it is evident that multiple causes for the deregulation of these genes must be considered in HCC, including non-traditional mechanisms.

## Introduction

The ubiquitin system is responsible for the post-translational modification of proteins, which mediates a range of processes, including proteasomal degradation (Hershko and Ciechanover, 1998; Bergink and Jentsch, 2009; Clague et al., 2015; Swatek and Komander, 2016; Rape, 2018). The sets of activating (E1), conjugating (E2), and ligating (E3) enzymes that come together to form ubiquitination complexes determine the varying mechanisms through which this system can affect protein stability, activity, interaction, and/or localization (Rape, 2018). Many alterations in this pathway—especially those affecting substrate-specific E3 ligases—have been implicated in cancer development (Hoeller and Dikic, 2009). Multi-omics analysis across 33 cancer types has found frequent upregulation of ubiquitin-proteasome pathway (UPP) genes, associated with a variety of genetic alterations, particularly DNA copy number (CN) alterations and hypomethylation (Ge et al., 2018). The significance of multi-omics analysis in HCC has been underscored by past demonstrations that mRNA expression, microRNA (miRNA) expression, CN variation, and DNA methylation collectively define distinct HCC subtypes and are predictive of survival (Liu et al., 2016; Chaudhary et al., 2018). Targeting the ubiquitin system for hepatocellular carcinoma (HCC) therapy would require a detailed understanding of the specific mechanisms of its disruption in the liver (Salami and Crews, 2017).

Cullin-RING E3 ubiquitin ligase (CRL) complexes are one of the best-known families of E3 ligases (Petroski and Deshaies, 2005). These complexes contain two subunits assembled on a Cullin protein scaffold: a RING-finger subunit, which recruits E2 enzymes, and a substrate-targeting subunit, which consists of an adaptor protein and a substrate-recognition protein (Sarikas et al., 2011; Wu et al., 2016). The CRL2^pVHL^ complex has been associated with renal cell carcinoma due to its von Hippel-Lindau (pVHL) tumour suppressor subunit (Hsieh et al., 2017). Under normoxic conditions, pVHL polyubiquitinates the hydroxylated form of the transcription factor hypoxia-inducible factor 1ɑ (HIF-1ɑ), leading to its degradation (Ivan et al., 2001). The proline hydroxylation of HIF-1ɑ is oxygen-dependent, and so in hypoxic conditions, pVHL does not ubiquitinate HIF-1ɑ, which can then dimerize with HIF-1β and activate genes involved in angiogenesis and other responses to hypoxia (Supplementary Figure S1) (Ivan et al., 2001). pVHL’s function is commonly eliminated in several types of cancer through deletions, loss-of-function mutations, or repressive methylation, which leads to ubiquitous HIF-1ɑ activation and the consequent stimulation of angiogenesis and anaerobic glycolysis (Gossage et al., 2015). CRL2^pVHL^ also consists of the scaffold protein Cullin-2 (CUL2), the RING-finger subunit Ring-Box 1 (RBX1), and the adaptor proteins Elongins B and C (ELOB and ELOC) (Supplementary Figure S1) (Kapitsinou and Haase, 2008; Cowey and Rathmell, 2009). When the complex is fully assembled, the ELOB/ELOC/pVHL subunit is bound to the N-terminal domain of CUL2, while RBX1 is bound to the C-terminal domain (Cardote et al., 2017). The CUL2 scaffold is highly flexible, which allows the ubiquitin-carrying E2 ligase recruited by RBX1 to interact with the substrate recruited by pVHL (Cardote et al., 2017).

Although CRL2^pVHL^ components are not traditionally considered oncogenes, they have been associated with cancer progression, often through interactions with alternative substrate-recognition subunits such as preferentially expressed antigen in melanoma (PRAME) (Jalava et al., 2009; Costessi et al., 2011; Okumura et al., 2012; Yang et al., 2013; Xu et al., 2015; Wang et al., 2016). MiRNAs have been implicated in the deregulation of CRL2^pVHL^ components in cancer; one example involves the downregulation of hsa-miR-194 in gastric cancer, which leads to RBX1 upregulation and subsequent increases in invasiveness (Chen et al., 2015). Downregulation of tumour-suppressing miRNAs allows for the upregulation of pro-tumour target genes, and is a known mechanism of deregulation in many cancer types (Zheng et al., 2012; Liao et al., 2014; Ge et al., 2018). Furthermore, the recent reporting of previously-undetected miRNAs in the human liver adds to the potential involvement of miRNAs in the dysregulation of genes in HCC, including UPP genes (Minatel et al., 2018).

While large-scale genomic profiling initiatives have uncovered several driver mutations for HCC, its highest-frequency alterations (e.g., *TP53* inactivating mutations, *CTNNB1* activating mutations) are not easily druggable (Cancer Genome Atlas Research Network, 2017). This lack of effective drug targets contributes to the poor 5-year survival rate of only 18% (Siegel et al., 2020). As such, uncovering novel and potentially-actionable alterations in liver cancer progression would be of significant clinical benefit. In this study, we investigate how each of the CRL2^pVHL^ component genes is deregulated in HCC. We interrogate genetic and epigenetic alterations that have frequently been associated with UPP gene overexpression in cancer, taking into consideration DNA CN, DNA methylation, and the expression of inhibitory miRNAs (Chan et al., 2011; Goto et al., 2016; Lee and Potts, 2017; Ge et al., 2018). We aim to investigate which of these events deregulate these genes, are associated with HCC, and impact patient survival.

## Methods

### Data Collection

HCC samples (*n* = 377) and adjacent non-malignant tissue (*n* = 59) were collected and processed by The Cancer Genome Atlas (TCGA) Research Network (http://cancergenome.nih.gov/). DNA CN, methylation, and gene expression data are publicly available and were retrieved from the TCGA Data Portal (accessed 28 February 2019). All non-synonymous somatic mutations were derived from exome sequencing generated on an Illumina HiSeq platform, and were obtained from the cBioPortal for Cancer Genomics (Cerami et al., 2012; Gao et al., 2013). Additionally, miRNA sequencing data were acquired from the Cancer Genomics Hub (CGHub) Data Repository (dbGaP Project ID: 6208). Clinical information was retrieved from the UCSC Xena Browser (https://xenabrowser.net/datapages/, accessed 9 June 2020).

Fetal liver samples (*n* = 10) were acquired in the form of anonymous pathological autopsy specimens from the Children’s and Women’s (C&W) Pathology laboratory at the BC C&W Hospital and Health Centre, following approval from the joint University of British Columbia/C&W Health Centre of British Columbia Research Ethics Board (H06-70085). Consent to the autopsies was given, and all identifying information was removed from samples. These samples were derived from elective terminations for medical reasons during the second trimester of gestation (17–24 weeks), and were chromosomally normal (46XX/46XY). Total RNA was isolated from frozen tissue using TRIzol (Life Technologies, Carlsbad, CA, United States). Small RNA sequencing was performed by Canada’s Michael Smith Genome Sciences Centre in Vancouver, on an Illumina HiSeq 2000 (Illumina, Inc., San Diego, CA, United States).

### Gene Expression Data Analysis

Fragments Per Kilobase of transcript per Million mapped reads (FPKM)-normalized mRNA data for the five CRL2^pVHL^ components were obtained from TCGA. FPKM values were log_2_ transformed, and mRNA overexpression was assessed based on the interquartile range (Tukey fence) method for detecting outliers. An HCC sample was considered to overexpress a given mRNA if its expression was greater than the upper Tukey fence for that mRNA in the non-malignant samples.

### Analysis of DNA Copy Number Alterations

CN alterations affecting the CRL2^pVHL^ component genes were calculated from TCGA-LIHC Affymetrix SNP 6.0 array data. Briefly, processed open access level 3 CN segment data were mapped to the GRCh38 (hg38) genome build using GDC Copy Number Liftover Workflow (Olshen et al., 2004; Beroukhim et al., 2010). CN variation was calculated using the GISTIC 2.0 algorithm, available at the GenePattern public server, using the following parameters: amp. threshold = 0.3, del. threshold = 0.3, confidence level = 0.75, qv threshold = 0.25, and broad length cutoff = 0.98 (Reich et al., 2006; Mermel et al., 2011). The GISTIC 2.0 algorithm provides discrete CN values of -2, -1, 0, 1 and 2, which represent homozygous deletion, single copy deletion, diploid normal copy, low-level CN amplification, and high-level CN amplification, respectively.

### Analysis of DNA Methylation Alterations

DNA methylation data (β values from the Illumina Infinium HM450K platform) were obtained from TCGA. β values were available for a total of 52 probes located within the CRL2^pVHL^ component genes, with at least eight for each component gene. Probes were considered hypomethylated in HCC relative to non-malignant liver if they had an average Δβ value < -0.1 and a *t*-test Benjamini-Hochberg-corrected *p* < 0.05. A gene was deemed hypomethylated in an individual HCC sample if the sample’s mean β value, considering only the HCC-hypomethylated probes within the gene in question, was below the lower Tukey fence for the mean β value of those probes in the non-malignant samples.

### MiRNA Quantification and Novel miRNA Discovery

Raw small RNA sequencing BAM files for both TCGA-LIHC and fetal liver samples were converted to unaligned (FASTQ) reads. Unaligned reads were then processed through the online platform miRMaster (Fehlmann et al., 2017). Briefly, miRMaster subjected sequence reads to adapter trimming, quality filtering, and read collapsing before alignment to the hg38 genome build. After alignment, reads that mapped to known miRNA loci in miRBase v21 were quantified. MiRMaster subsequently input unmapped reads into an AdaBoost-based classifier that had been trained to identify genuine miRNA precursors, using a feature set that includes measures of free energy, folding, and nucleotide abundance. A predicted miRNA precursor was only considered novel by miRMaster if it did not overlap with a miRBase entry, and contained no annotated miRNAs. Annotated (“known”) and novel miRNAs were both filtered by expression level in each tissue type (≥ 1 RPM in ≥ 10% of samples and > 0 RPM in ≥ 50% of samples). Known and novel miRNA expression data for the fetal liver samples have been deposited in the Gene Expression Omnibus (GSE194052) (Lam, 2022).

### Analysis of miRNA Differential Expression

A Wilcoxon signed-rank test was applied to identify both annotated and novel miRNAs that were differentially expressed between paired HCC and non-malignant tissues (*n* = 49 pairs). MiRNAs were classified as differentially expressed if they had a Benjamini-Hochberg-corrected *p* value < 0.05, and a median expression > 0 RPM in one or both of the tumour and non-malignant sample groups.

For the purpose of survival analysis, it was necessary to identify exactly which HCC samples displayed downregulation of a given miRNA, and which did not. An HCC sample was considered to have downregulation of a given miRNA if its z-score for that miRNA’s expression, with respect to expression in the non-malignant samples, was < −1.645, corresponding to the bottom 5% of the distribution.

### MiRNA Target Prediction and Correlation with Gene Expression

The predicted mRNA targets of all annotated, HCC-downregulated miRNAs were retrieved from the mirDIP 4.1 database (Tokar et al., 2018). Only miRNA-mRNA target interactions that received integrative scores in the 99th percentile were considered for further analysis. Target prediction for novel miRNAs was performed using the miRanda v3.3a algorithm with strict alignment, requiring an alignment score ≥ 140 and with an energy threshold of ≤ −20 kcal/mol (Enright et al., 2003). Mature miRNA sequences were queried against the 3′-UTR sequences of all human genes, obtained from Ensembl using BioMart (Kinsella et al., 2011). Spearman’s correlation test was used to identify miRNAs whose expression was negatively correlated with that of one or more CRL2^pVHL^ component genes, using the thresholds ρ < -0.2 and *p* < 0.05.

### Multidimensional Scores and Survival Analyses

For each HCC patient, a multidimensional score was calculated for each CRL2^pVHL^ gene, with higher scores indicating a greater number of genetic or epigenetic alterations to that gene. All scores started at zero. In a given patient, a gene’s score was increased by one point for each of the following features: mRNA upregulation, downregulation of at least half of the annotated miRNAs that were predicted to target the gene, CN increase, and hypomethylation.

For all survival analyses, differences in outcome between patient groups were considered significant if *p* < 0.05, as given by a log-rank test (in the case of two groups) or a log-rank test for trend (in the case of three or more groups). Data from >2000 days was included when performing statistical tests. All Kaplan-Meier plots were generated using MatSurv (Creed et al., 2020).

### Software Used for Data Analysis and Visualization

SPSS v. 21.0, MATLAB R2017b (MathWorks), GraphPad Prism v. 5.0, and RStudio v. 0.98.484 were used to perform statistical analysis and generate figures. The ComplexHeatmap R package (Gu et al., 2016) and the Gene Structure Display Server v. 2.0 (Hu et al., 2015) were also used for figure generation.

## Results

### The CRL2^pVHL^ Complex is Altered Across Cancer Types

The genes coding for CRL2^pVHL^ components are known to be frequently altered in cancer, particularly the tumour suppressor gene *VHL* in kidney cancers, and have been previously associated with cancer progression. To gain a pan-cancer perspective on alterations to these genes, we evaluated the DNA CN status of *VHL*, *CUL2*, *RBX1*, *ELOC*, and *ELOB* across 33 cancer types processed by TCGA. Shallow deletion of *VHL* was prevalent in kidney clear cell carcinoma (76.7%), but substantially less common in other cancer types collectively (23.7%) (Supplementary Figure S2). ELOC was the most commonly altered component across all cancer types, and was frequently gained (35.4%). Notably, gains and amplifications in *ELOC* were prevalent in head and neck squamous cell carcinoma (63.8%) and HCC (55.7%) (Supplementary Figure S2).

### CRL2^pVHL^ Component Expression is Associated With HCC Patient Outcome

**FIGURE 1:**
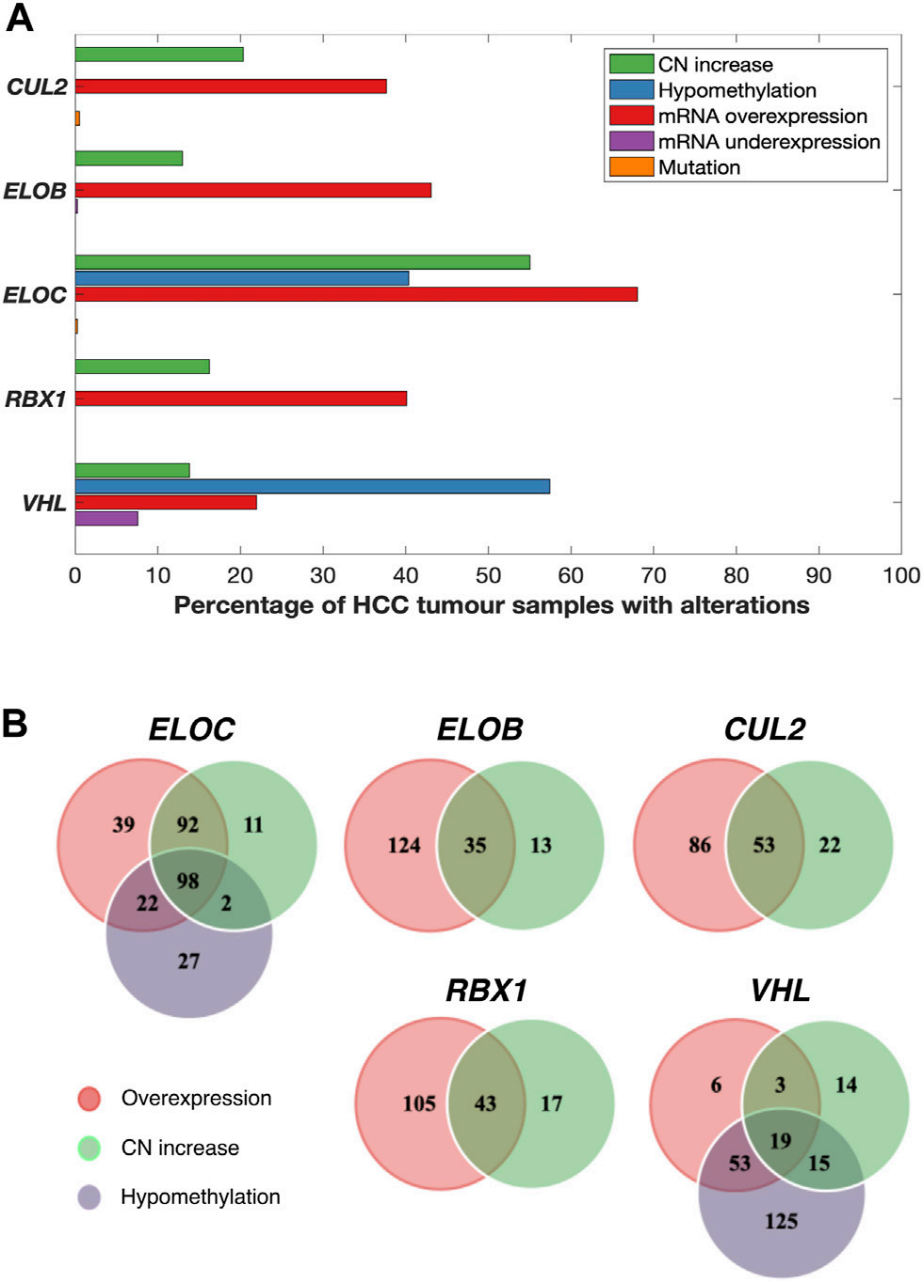
Various combinations of mRNA upregulation, copy number increase, and hypomethylation alterations are common in CRL2^pVHL^ component genes in HCC. **(A)** The bar graph indicates the prevalence of alterations to the CRL2^pVHL^ component genes in TCGA-LIHC tumours for which complete data were available (*n* = 369). CN (copy number) increase includes both genomic gain and amplification. **(B)** The Venn diagrams display the numbers of HCC samples that harbor overlapping and/or unique alterations in each CRL2^pVHL^ component gene.

**FIGURE 2:**
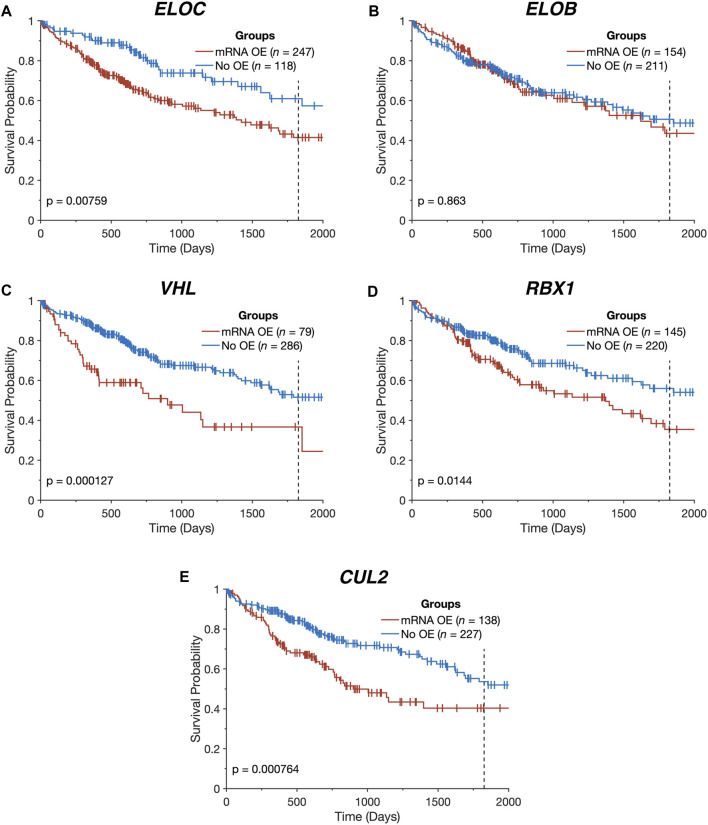
Overexpression of CRL2^pVHL^ component genes is linked to worse overall survival. **(A–E)** Kaplan-Meier plots indicating the overall survival over time of TCGA-LIHC patients (*n* = 365), stratified by the presence of mRNA overexpression (OE) for each CRL2^pVHL^ component gene. An mRNA was deemed overexpressed in a given HCC sample if its expression exceeded the upper Tukey fence for that mRNA in the non-malignant samples. Plots are cut off at 2,000 days, and the 5-year mark is denoted by a dotted line.

All of the CRL2^pVHL^ component genes were frequently upregulated in TCGA-LIHC tumour samples in comparison to adjacent non-malignant tissue, particularly *ELOC* (68.0% of HCC tumours) and *ELOB* (43.1%) (Figures 1A,B). However, unlike in renal cell carcinoma, only 28 out of 369 cases (7.6%) displayed *VHL* mRNA downregulation (Figure 1A). In order to evaluate whether the upregulation of CRL2^pVHL^ complex genes influences HCC patient outcome, we stratified HCC patients by their mRNA expression of each complex component, and examined the difference in outcomes between patient groups. Overexpression of *VHL*, *CUL2*, *ELOC*, or *RBX1* was found to be associated with significantly worse patient outcomes (*p* < 0.05) (Figures 2A–E).

### Both Genetic and Epigenetic Mechanisms Affect CRL2^pVHL^ Component Gene Expression

**FIGURE 3:**
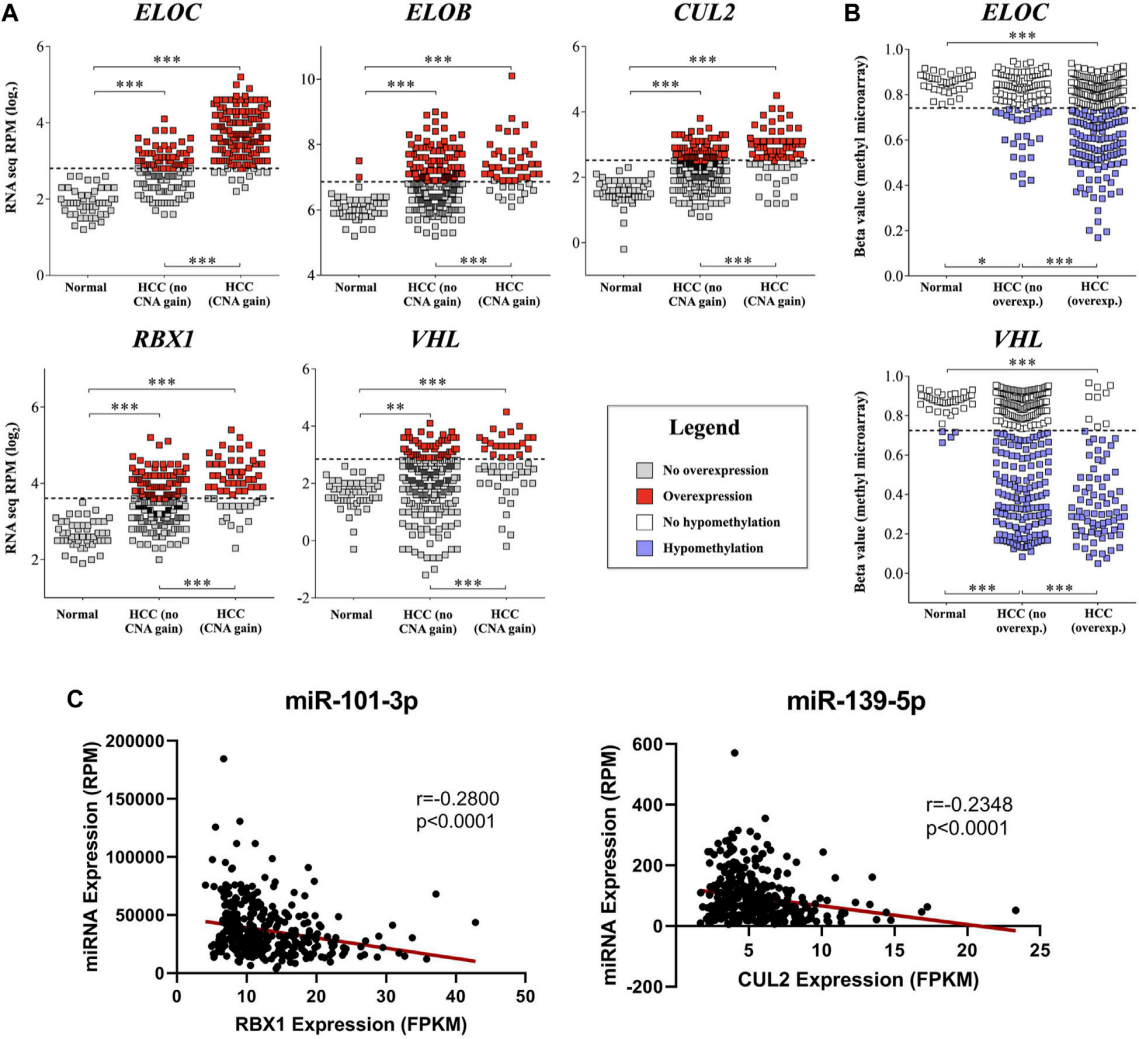
mRNA upregulation of CRL2^pVHL^ component genes is correlated with copy number increase, hypomethylation, and miRNA downregulation. **(A)** Comparisons of mRNA expression of CRL2^pVHL^ component genes between non-malignant samples and HCC samples, where the latter are stratified by the presence of CN (copy number) increase. Dashed lines represent the upper Tukey fence of the non-malignant group, and were considered the threshold of overexpression; **(B)** Comparison of component gene methylation between non-malignant samples and HCC samples, where the latter are stratified by the presence of gene overexpression. Dashed lines represent the lower Tukey fence of the non-malignant group, and were considered the threshold of hypomethylation. **p < 0.01; ***p < 0.001; **(C)** Examples of negative correlations between the mRNA expression of component genes and the expression of HCC-downregulated annotated miRNAs that were predicted to target at least one component gene.

In order to understand the mechanisms associated with mRNA upregulation of CRL2^pVHL^ component genes in HCC, we sought to evaluate the association between possible mechanisms and mRNA upregulation in a case-by-case manner. The only components affected by mutations were CUL2 and ELOC, and at very low frequencies (0.5% and 0.3%, respectively) (Figure 1A). CN segment data revealed that CN increase of each of the component genes was common in HCC, as each gene was either gained or amplified in at least 10% of cases (Figures 1A,B). For each component gene except *VHL*, at least 70% of cases harboring CN gain or amplification also displayed mRNA upregulation (Figure 1B). There was a significant correlation between mRNA upregulation and CN increase for all of the individual component genes, suggesting a contribution of CN increase to mRNA upregulation (Figure 3A).

We next assessed the methylation status of the component genes, as changes in methylation are also a well-characterized mechanism of mRNA regulation. Methylation data were available for a total of 52 probes located within the component genes, of which three were hypomethylated in HCC relative to non-malignant liver tissue samples: one within the 5′-UTR of *ELOC* (cg17316966), and two within the gene body of *VHL* (cg16869108 and cg20916523) (Supplementary Figure S3). Hypomethylation at these sites was associated with increased expression of *ELOC* and *VHL* (Figures 1B, 3B; Supplementary Figure S3). Of 251 cases with *ELOC* upregulation, 22 (9%) displayed hypomethylation but not CN increase, suggesting that hypomethylation can independently contribute to *ELOC* upregulation (Figure 1B). Notably, 53 of 81 cases (65%) with *VHL* upregulation were found to have hypomethylation without CN increase, as opposed to only 3 cases (4%) with CN increase in the absence of hypomethylation (Figure 1B).

Consequently, we postulate that hypomethylation is a major mechanism of *VHL* upregulation and also a contributing factor in *ELOC* upregulation, whereas CN increase is a major mechanism of *CUL2*, *RBX1*, *ELOB*, and *ELOC* upregulation in HCC.

### MiRNA-Mediated Deregulation of the CRL2^pVHL^ Component Genes

Although significant association between mRNA upregulation and CN increase or hypomethylation was found for all of the CRL2^pVHL^ component genes, many cases of mRNA upregulation occur in the absence of those two mechanisms (Figure 1B). One possible explanation is that miRNAs that target component gene mRNAs are downregulated in these cases. In order to assess this potential mechanism, we first obtained the broadest possible view of the liver miRNA transcriptome by identifying all miRNAs that were expressed in HCC, adjacent non-malignant tissues, or an independent set of fetal liver samples. Out of the total of 859 expressed miRNAs, 276 were downregulated in HCC samples relative to paired non-malignant liver samples (Supplementary Table S1). To investigate how these miRNAs might impact HCC cell biology, we performed mRNA target prediction and found that 27 (10%) of these downregulated miRNAs were predicted to target at least one of the CRL2^pVHL^ component genes (Supplementary Table S2).

Additionally, we found that the expression of five of these 27 miRNAs correlated negatively with that of at least one CRL2^pVHL^ component gene (Spearman’s ρ < -0.2, *p* < 0.0001) (Supplementary Table S3). Both miR-101-3p and miR-139-5p were predicted to target *ELOC*, and were found to be negatively correlated with three and four of the component genes, respectively (Figure 3C, Supplementary Figure S4). Of the 27 miRNAs, above-median RPM expression levels of miR-101-3p, miR-139-5p, or miR-497-5p were each associated with improved overall survival (Supplementary Table S4). These results evidence that miRNA-mediated deregulation of CRL2^pVHL^ component genes plays a role in HCC biology.

### Potential Role of Previously-Undetected miRNAs in CRL2^pVHL^ Component Gene Deregulation

We next investigated whether downregulation of miRNAs that have not yet been annotated in miRBase (v21) might also contribute to CRL2^pVHL^ regulation. Using a previously described discovery pipeline (Fehlmann et al., 2017), we identified 309 novel miRNAs in the samples, of which 106 were downregulated in HCC relative to paired non-malignant tissue (Supplementary Table S5). These 106 novel miRNAs are similar to the 276 downregulated known miRNAs in their average length and GC content, as well as their average novoMiRank score, which is a measure of similarity to miRNAs catalogued in early versions of miRBase (Backes et al., 2016) (Supplementary Table S6).

Of the 106 downregulated novel miRNAs, 39 (37%) were predicted to target at least one CRL2^pVHL^ component gene. Additionally, significant negative correlation with at least one CRL2^pVHL^ component gene was found for 10 of the 106 novel miRNA candidates (Spearman’s *ρ* < −0.2, *p* < 0.0001) (Supplementary Table S7). Several of these 10 previously unannotated miRNAs were negatively correlated with multiple complex components, including LIHC-nov-miR-41 and LIHC-nov-miR-133 (Supplementary Table S7, Supplementary Figure S5). The former was negatively correlated with all of the component genes except *ELOB*, highlighting the potential regulatory ability of novel miRNAs on the CRL2^pVHL^ complex. Additionally, the RPM expression levels of two of these 10 novel miRNAs (LIHC-nov-miR-133 and LIHC-nov-miR-112) were significantly associated with overall survival (Supplementary Table S8).

### Impact of Alterations Affecting CRL2^pVHL^ Component Genes on Patient Outcome

**FIGURE 4:**
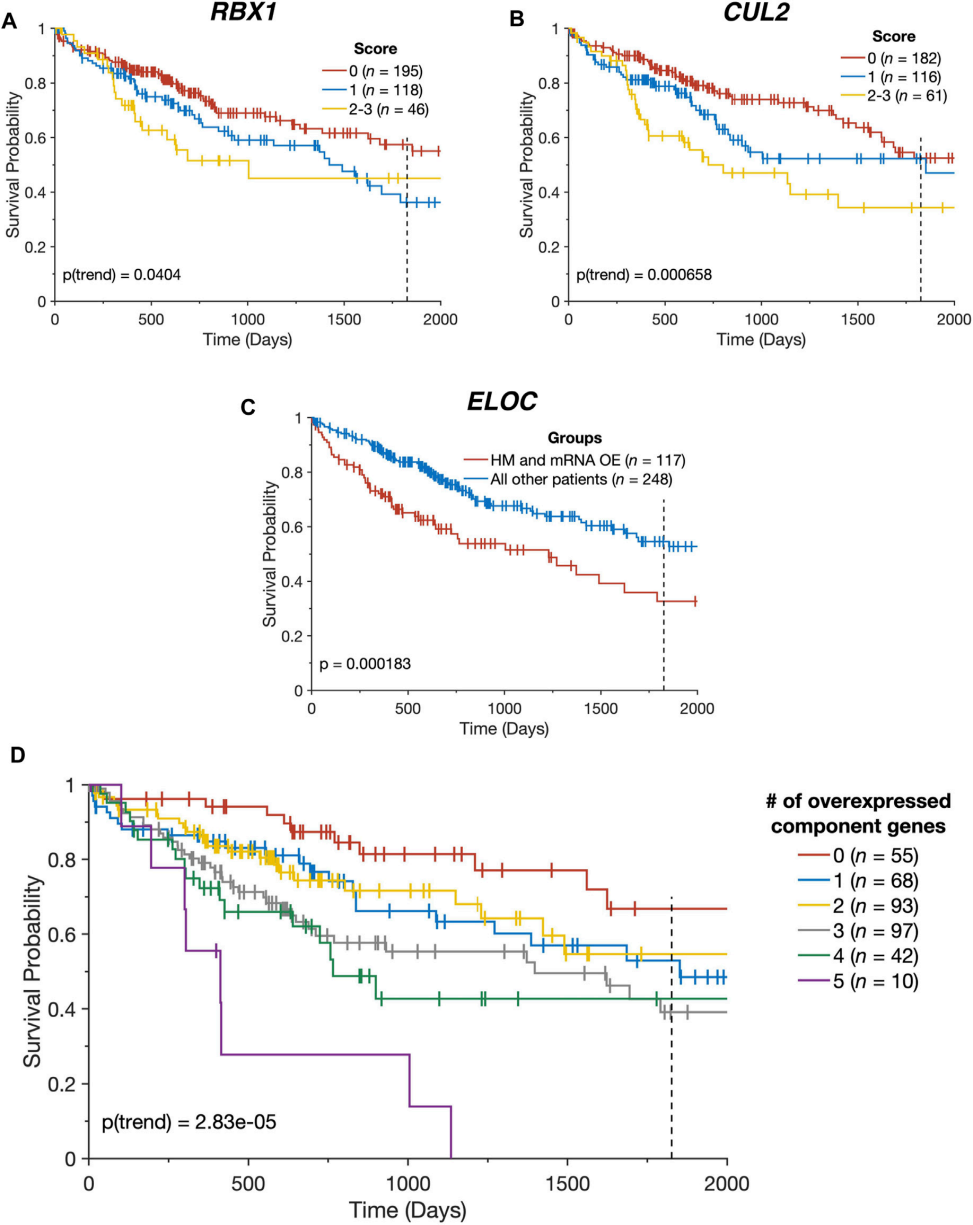
CRL2^pVHL^ component gene alterations impact patient outcomes. **(A,B)** Kaplan-Meier plots indicating the overall survival over time of TCGA-LIHC patients, stratified by the number of alterations each had affecting the specified gene (*n* = 359). Groups containing five or fewer patients were combined with the nearest larger group; **(C)** Kaplan-Meier plot indicating the overall survival over time of TCGA-LIHC patients (*n* = 365), divided between patients with both *ELOC* hypomethylation (HM) and mRNA overexpression (OE), and all other patients; **(D)** Kaplan-Meier plot indicating the overall survival over time of TCGA-LIHC patients (*n* = 365), stratified by the number of CRL2^pVHL^ component genes with mRNA overexpression. For all panels, plots are cut off at 2000 days and the 5-year mark is indicated by a dotted line.

In light of the combined contributions of CN alterations, DNA hypomethylation, and miRNA downregulation to the upregulation of the CRL2^pVHL^ component genes, we examined the collective impact of these various alterations on patient survival. We constructed a multidimensional score that represents the number of alterations (mRNA overexpression, CN increase, hypomethylation, and miRNA downregulation) a patient has that affect a given gene. For *RBX1* and *CUL2*, we observed that patients with greater numbers of alterations tended to experience poorer overall survival (*p* = 0.04 and *p* = 6.58 × 10^−4^, respectively) (Figures 4A,B). Additionally, patients with both *ELOC* overexpression and hypomethylation experienced significantly worse outcomes than those with only one or neither alteration (*p* = 1.83 × 10^−4^) (Figure 4C). This division of patients stratified patients by survival with greater efficacy than a division based on *ELOC* overexpression alone (*p* = 1.83 × 10^−4^ and *p* = 7.59 × 10^−3^, respectively) (Figures 2A, 4C). Finally, there was a significant tendency for patients with mRNA upregulation of a greater number of CRL2^pVHL^ component genes to experience poorer overall survival than those with upregulation of few or none of those genes (*p* = 2.83 × 10^−5^), suggesting that these alterations are non-redundant (Figure 4D).

## Discussion

Ubiquitin pathway genes are known to be frequently altered in tumours, and many have been implicated in cancer development. CRL2^pVHL^ is a well-known ubiquitin ligase complex due to its frequent inactivation in kidney cancers, but its role in the development of other cancer types has been largely unexplored. Here, we demonstrate that all component genes of the CRL2^pVHL^ complex are upregulated in HCC; that copy number increase, hypomethylation, and miRNA downregulation contribute to this upregulation; and that this upregulation is linked to decreased patient survival. This association between CRL2^pVHL^ upregulation and HCC development is counter-intuitive, as CRL2^pVHL^ is known to ubiquitinate and encourage the degradation of HIF-1ɑ (Maxwell et al., 1999; Ke and Costa, 2006), suggesting that CRL2^pVHL^ overexpression would lead to an inability to overcome hypoxia. CRL2^pVHL^ has also been implicated in the degradation-promoting ubiquitination of other substrates, but many of them, including activated epidermal growth factor receptor (EGFR), zinc fingers and homeoboxes 2 (ZHX2), and never-in-mitosis A-related kinase 8 (NEK8), have also been linked to tumour growth or poor patient outcomes (Zhou and Yang, 2011; Ding et al., 2018; Zhang et al., 2018).

pVHL is the least-frequently upregulated component of the CRL2^pVHL^ complex in HCC. This is likely because of its traditional role as a tumour suppressor. In other cancers, it is predominantly affected by deactivating mutations, but it can also be epigenetically silenced through hypermethylation of its promoter in tumours, particularly in clear cell renal cell carcinoma (Herman et al., 1994; Cancer Genome Atlas Research Network, 2013). Counter to this, we observed that a large proportion of HCC samples displayed hypomethylation at sites within the *VHL* gene body, and many had mRNA upregulation. Still, the well-established tumour suppressive function of pVHL suggests that pVHL-independent functions of the other complex components may instead be driving HCC aggressiveness. For instance, CUL2, RBX1, ELOB, and ELOC are known to interact with PRAME, a substrate-recognition subunit that is overexpressed in a variety of cancers, including HCC (Costessi et al., 2011; Zhu et al., 2018). Additionally, PRAME expression is associated with poor prognosis, likely due to inhibiting the activity of the retinoic acid signaling response (Costessi et al., 2011). Furthermore, both CUL2 and RBX1 have been shown to contribute to the ubiquitination and degradation of the tumour suppressor RHOB in liver cancer (Xu et al., 2015). Knockdown of *RBX1* has also been correlated with decreases in the growth of HCC xenografts and the induction of cell cycle arrest and senescence in HCC cells (Yang et al., 2013). RBX1, ELOB, and ELOC can also interact with CUL5 and WSB1, both of which are overexpressed in HCC, to form the CRL5^WSB1^ complex (Okumura et al., 2012; Xu et al., 2020; Li et al., 2021). This E3 ligase complex has a number of targets, including HIPK2, whose loss downregulates apoptosis, and pVHL (Okumura et al., 2012; Kim et al., 2015). In this way, overexpression of CRL5^WSB1^ could promote tumour development by allowing cells to resist pro-apoptotic signaling and protecting HIF-1ɑ from pVHL-mediated degradation. Hence, the CRL2^pVHL^ components may participate in both pro- and anti-tumour functions. While the kidney is highly dependent on tumour suppressive pVHL function, the work presented here suggests that liver biology is more influenced by the tumour-promoting functions of the other complex components than previously known, meriting further study.

Although genomic CN gain or amplification and hypomethylation are common mechanisms of CRL2^pVHL^ upregulation in HCC, we found that the downregulation of miRNAs that target CRL2^pVHL^ component mRNAs may also play a significant role. We unearthed 27 annotated miRNAs and 39 novel miRNA candidates that are downregulated in liver tumour samples when compared to adjacent non-malignant tissues, and that are predicted to target at least one of the CRL2^pVHL^ complex genes. Furthermore, significant negative correlation between each of 15 miRNAs, including 10 novel miRNAs, and the mRNA expression of at least one CRL2^pVHL^ component gene was observed. As miRNAs can also function through the translational inhibition of target genes, we would not expect to see a negative correlation between all miRNAs and their respective targets.

In particular, LIHC-nov-miR-41 was significantly negatively correlated with the expression of four of the five CRL2^pVHL^ component genes. The likely involvement of novel miRNAs in CRL2^pVHL^ regulation supports the notion that traditional miRNA detection methods, which have predominantly focused on highly-expressed sequences that are conserved between tissues, have overlooked many significant tissue-specific miRNAs (Londin et al., 2015). In fact, unannotated miRNAs with distinct expression patterns in each of the thyroid and head and neck, among other tissues, have previously been discovered, and show the potential to be involved in tumour development and impact prognosis (Barros-Filho et al., 2019; Rock et al., 2019). Similarly, it was previously shown that fetal samples are sources of novel miRNAs (Zhao et al., 2015), and that expression of fetal miRNAs can also be a mechanism driving cancer (Ma et al., 2012; Becker-Santos et al., 2016). Interestingly, we found that several of the miRNAs predicted to target CRL2^pVHL^ component genes, such as miR-101, were expressed in fetal liver samples.

Both miR-101-3p and miR-139-5p were found to be negatively correlated with a majority of the five CRL2^pVHL^ component genes. MiR-101-3p is known to be significantly downregulated in HCC, and has been recognized as a potential diagnostic marker and tumour suppressor (Su et al., 2009; Shen et al., 2014; Li et al., 2017). Low expression of miR-101-3p impacts prognosis, as it is correlated with later TNM stages, high alpha-fetoprotein levels, and poor overall survival (Budhu et al., 2008; Li et al., 2017). Low levels of mir-139 have been associated with poor outcomes in HCC as well (Hua et al., 2018). In contrast, overexpression of miR-139-5p has been shown to curtail aerobic glycolysis in HCC cells and to diminish their capacity for invasion, migration, and proliferation (Hua et al., 2018). Together, this suggests that miR-101-3p and miR-139-5p oppose the development of liver tumours, possibly through their regulation of the CRL2^pVHL^ complex. Additionally, miR-214-3p and miR-195-5p, which were predicted to target one or more component genes, have been previously shown to be downregulated in HCC, and to target β-catenin and downstream NF-κB effectors, respectively (Su et al., 2009; Shi et al., 2015). Our findings suggest that in addition to their roles in regulating these important cancer pathways, they may also contribute to CRL2^pVHL^ regulation (Su et al., 2009; Shi et al., 2015).

The combined significance of mRNA overexpression, CN increase, hypomethylation, and miRNA downregulation is further highlighted by our finding that patients with increased numbers of these alterations in *RBX1* or *CUL2* experience poorer outcomes. This further supports the notion that the deregulation of the CRL2^pVHL^ complex in HCC is not due to a single common cause, but rather several causes that can compound to affect patient outcomes. The multimodal nature of this regulation has also been observed in HCC more generally, where models that incorporate data on CN variations, hypomethylation, and miRNA expression more accurately predict mRNA expression than those that discard any individual mode (Kazan, 2016). Similar forms of multimodal deregulation have also been observed in oncogenes outside of the HCC context, including *EGFR*, *MDM2*, and *PDGFRA* (Louhimo and Hautaniemi, 2011), as well as other UPP genes (Ge et al., 2018). A more thorough understanding of the less-studied potential causes of CRL2^pVHL^ deregulation, such as the downregulation of previously-unannotated miRNAs, is essential to understanding dysfunctions of the ubiquitin-proteasome system in patients without CN alterations or hypomethylation.

The ubiquitin-proteasome system is frequently altered across all cancer types, and targeting it is a compelling therapeutic strategy for tumours whose most prevalent driver mutations are not easily druggable. The upregulation of CRL2^pVHL^ components seen here in HCC could make them potent targets, but the complete abrogation of their activity would likely lead to tumour-supporting HIF-1ɑ activation. A deeper understanding of the various functions of each CRL2^pVHL^ complex component may allow for the development of specific inhibitors that attenuate their individual pro-tumour activities without impacting the complex’s ability to regulate HIF-1ɑ.

## Data Availability

The datasets presented in this study can be found in online repositories. The names of the repository/repositories and accession number(s) can be found in the article/Supplementary Material.
