# Supplementary material for: Genetic and Epigenetic Mechanisms Deregulate the CRL2pVHL Complex in Hepatocellular Carcinoma
Source: Front Genet. 2022 May 18;13:910221. doi: 10.3389/fgene.2022.910221 (PMC9159809; doi:10.3389/fgene.2022.910221)
Supplement: Supplementary file 1 [file DataSheet1.DOCX]

Supplementary Material

# Supplementary Figures


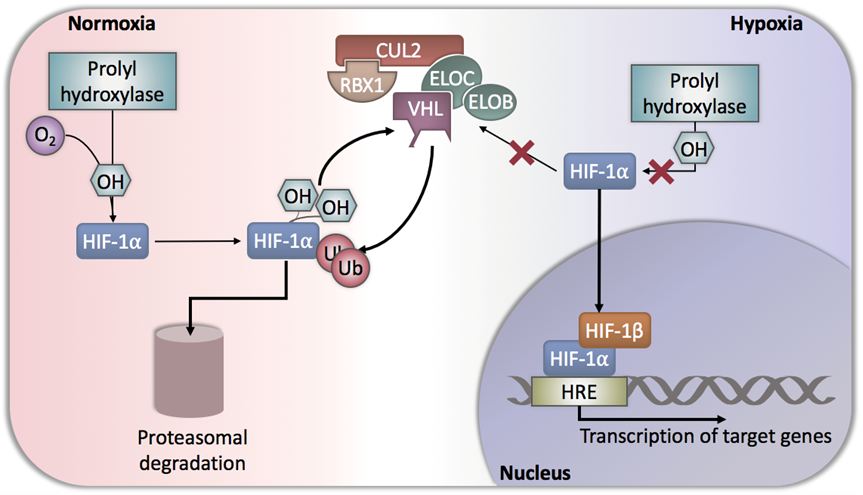


**Supplementary Figure 1.** Schematic of the regulation of HIF-1ɑ by the CRL2^pVHL^ complex. In normoxic conditions, two proline residues within HIF-1ɑ are hydroxylated by oxygen-dependent prolyl hydroxylase enzymes. Hydroxylated HIF-1ɑ is then bound by pVHL, in complex with ELOC, ELOB, CUL2, and RBX1. pVHL polyubiquitinates HIF-1ɑ, marking it for degradation by the proteasome. When oxygen levels are low, HIF-1ɑ is not hydroxylated, and is consequently not recognized by pVHL. HIF-1ɑ translocates to the nucleus, dimerizes with HIF-1β, and binds to hypoxia response elements (HREs) in target genes, promoting their transcription and the activation of angiogenic and glycolytic pathways.

#
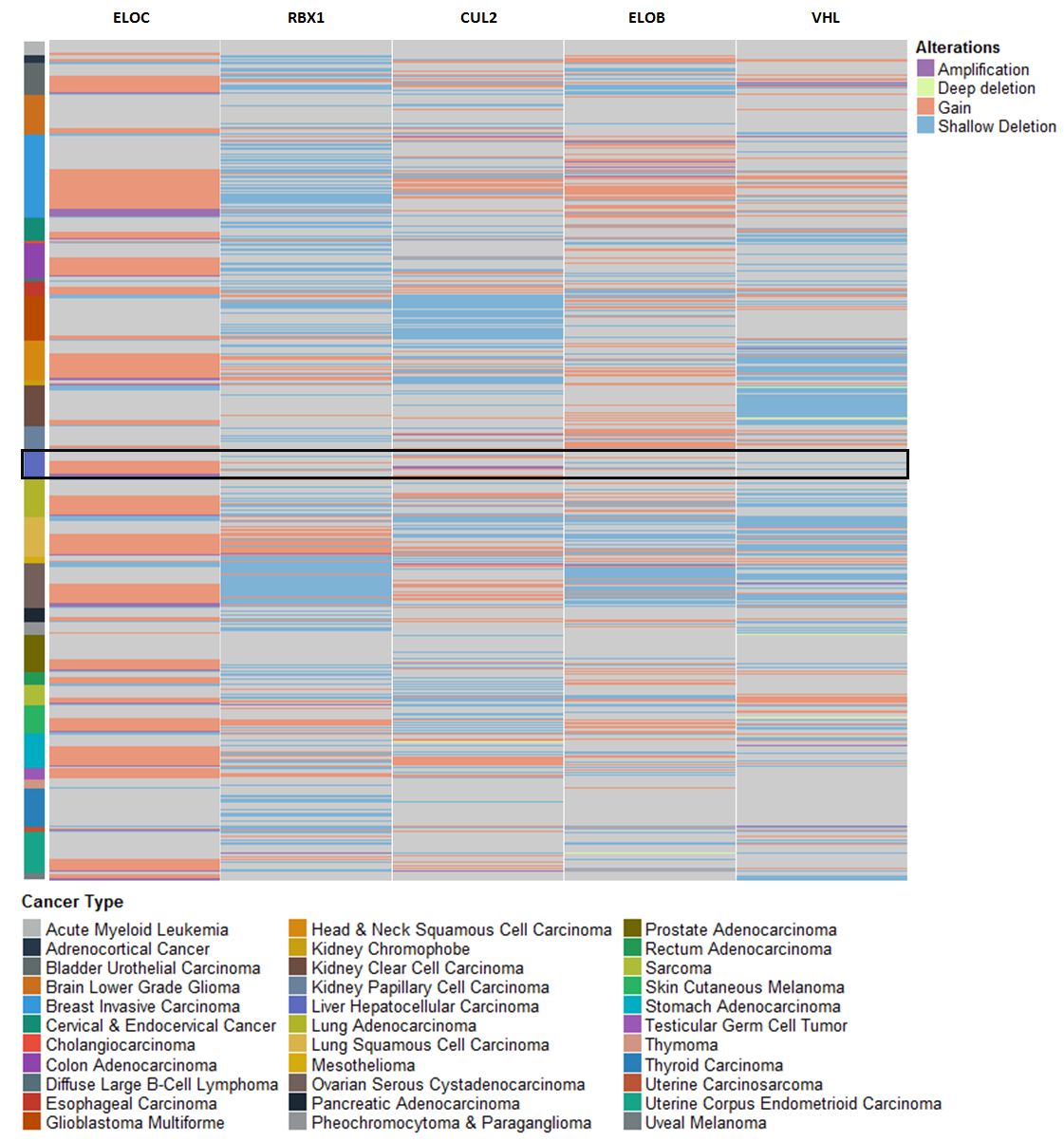


**Supplementary Figure 2.** Overview of CRL2^pVHL^ copy number alterations in 33 TCGA cancer types. Each row represents a patient tumour, with the coloured panel on the left indicating the cancer type, with reference to the legend at the bottom. The ComplexHeatmap R package (47) was used to generate this image.

**
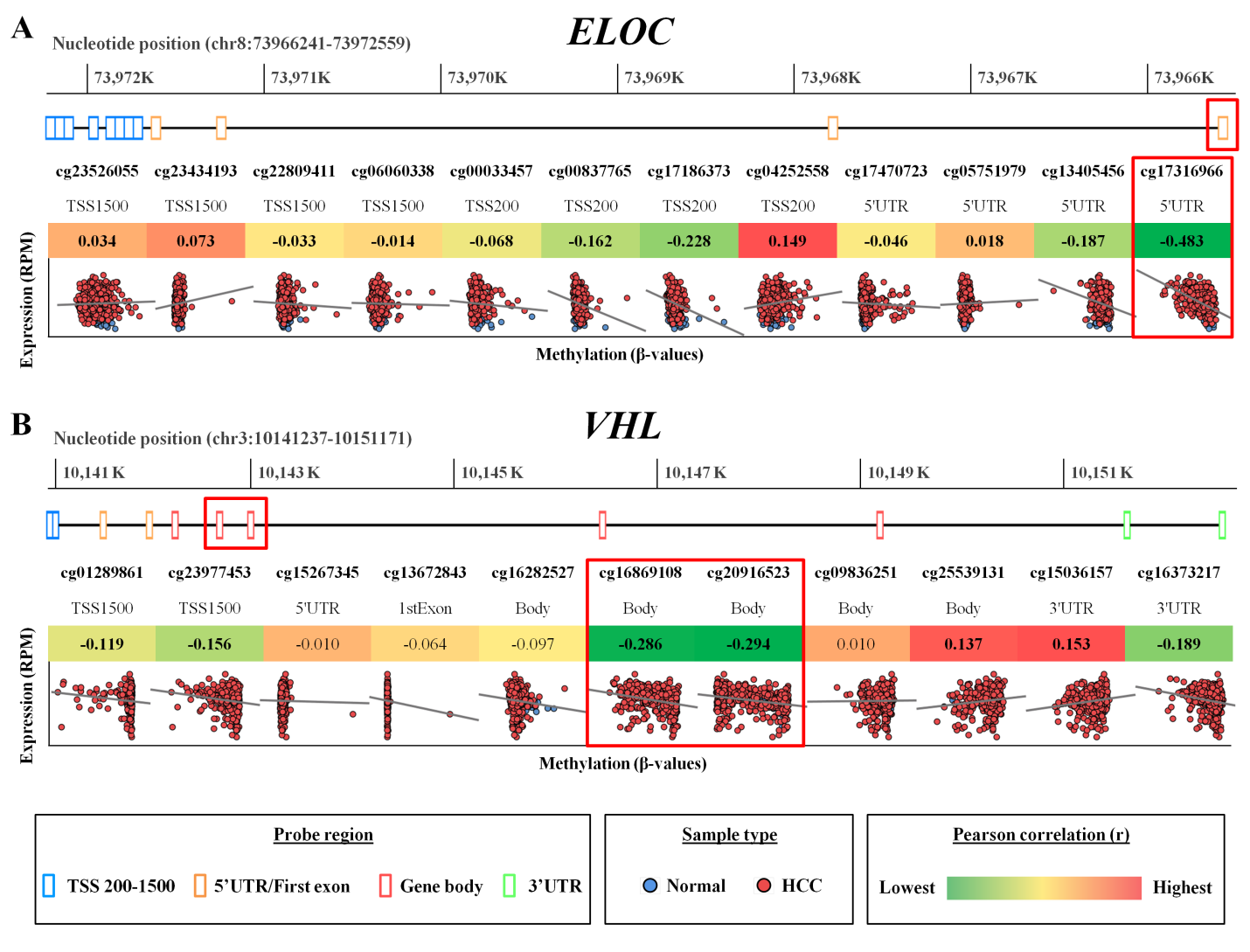
**

**Supplementary Figure 3.** Identification of HCC-hypomethylated probes in *VHL* and *ELOC*. Data for all methylation probes (Illumina Infinium Human Methylation 450k platform) within the genomic regions of *ELOC* **(A)** and *VHL* **(B)** are shown, as obtained from the TCGA Data Portal. Scatter plots display the relationship between probe methylation and gene expression, with lines of best fit superimposed and Pearson correlation coefficients (r) indicated. Probes deemed to be hypomethylated in HCC relative to adjacent non-malignant tissue are outlined in red, and have the following genomic locations: cg17316966 – chr8:73966241-73966242; cg16869108 – chr3:10142635-10142636; cg20916523 – chr3:10142900-10142901. The Gene Structure Display Server v. 2.0 (48) was used in figure generation.

**
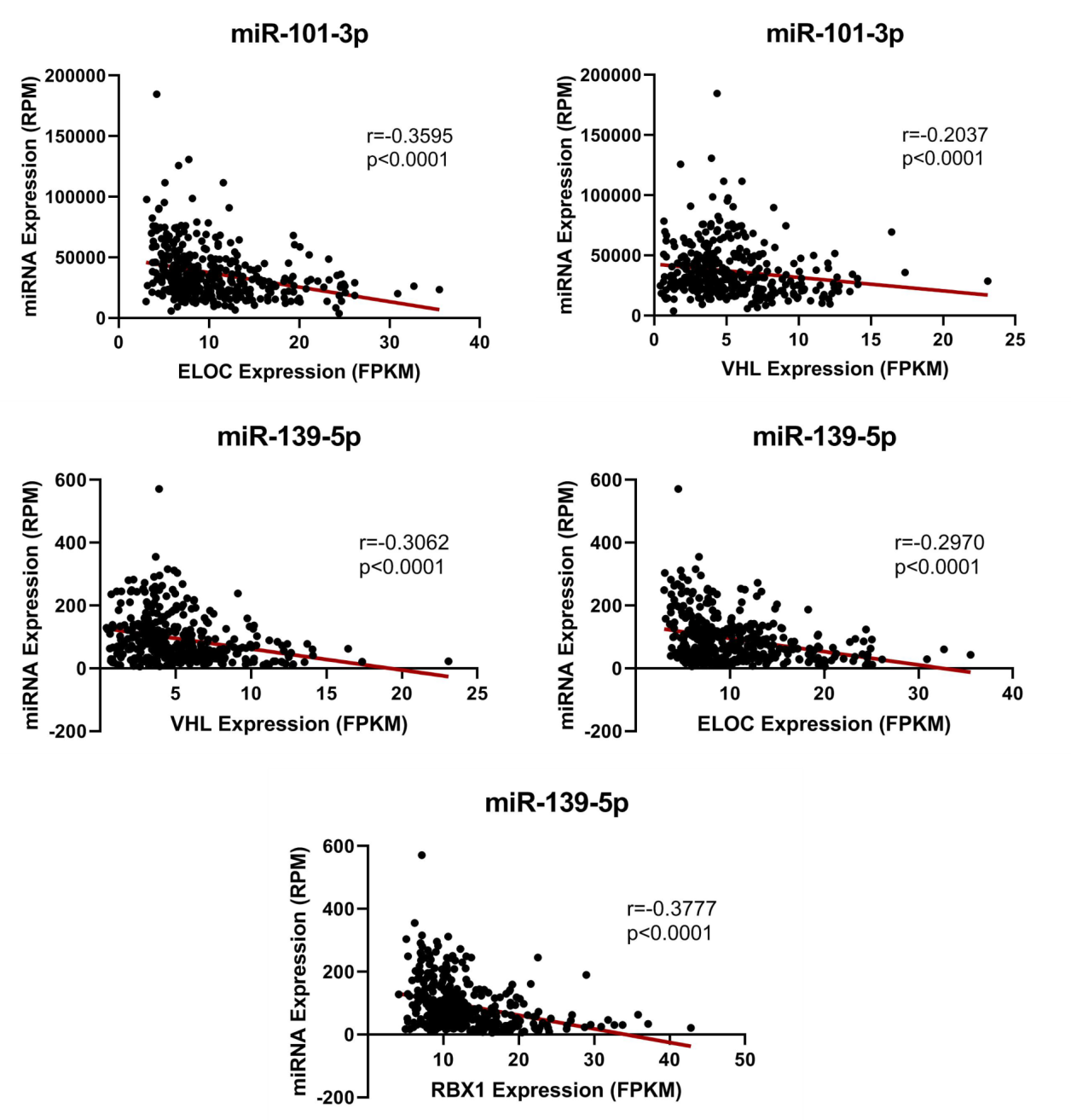
**

**Supplementary Figure 4.** Expression of CRL2^pVHL^ component genes is correlated with that of certain annotated miRNAs predicted to target them. Scatter plots display examples of negative correlations between the expression of mRNAs of CRL2^pVHL^ component genes and HCC-downregulated annotated miRNAs that were predicted by mirDIP 4.1 to target at least one component gene. Significance was assessed using Spearman’s correlation test (ρ < -0.2 and *p* < 0.05).


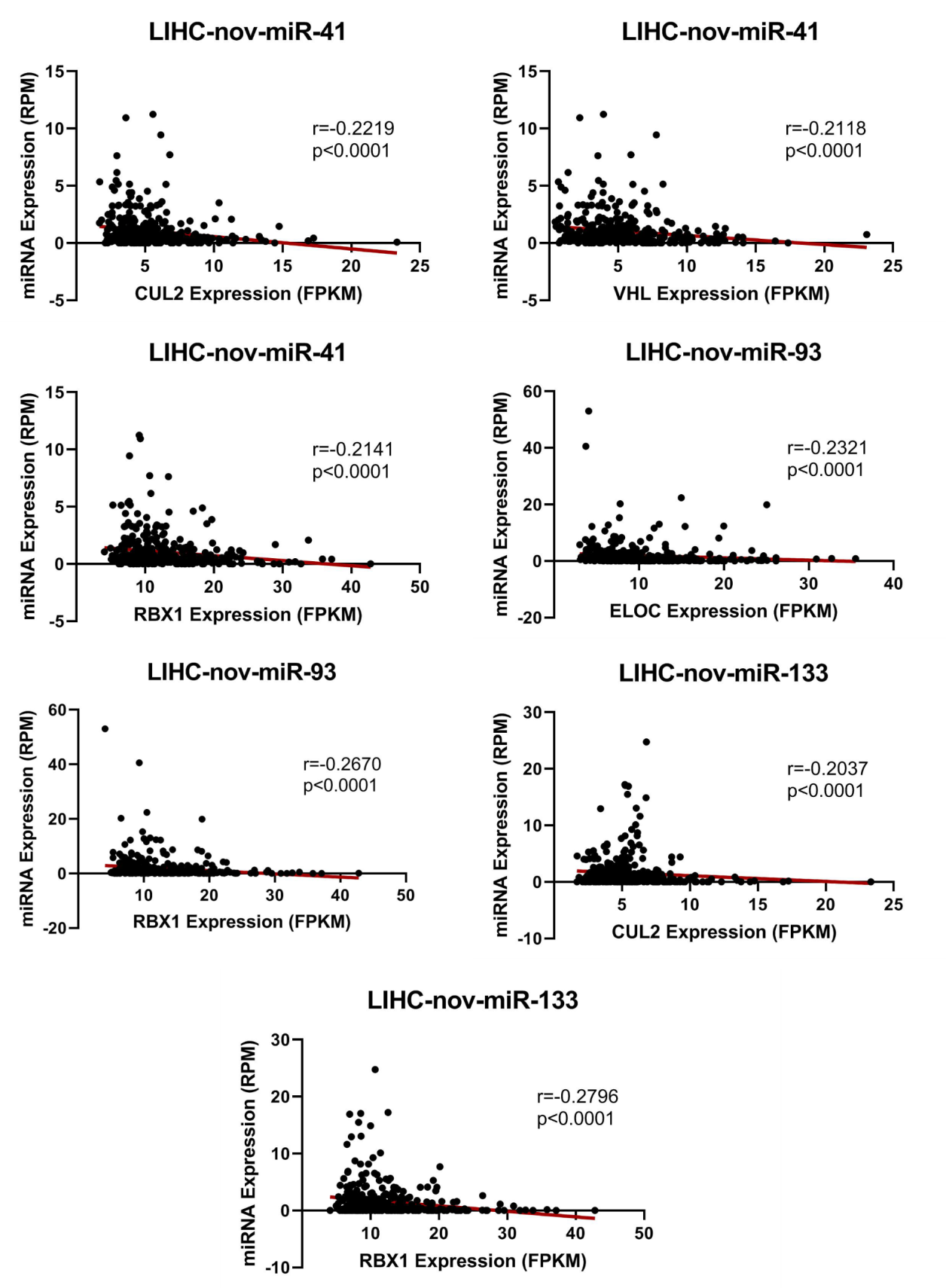


**Supplementary Figure 5.** Expression of CRL2^pVHL^ component genes is correlated with that of certain novel miRNAs. Scatter plots display examples of negative correlations between the expression of mRNAs of CRL2^pVHL^ component genes and HCC-downregulated novel miRNAs. Significance was assessed using Spearman’s correlation test (ρ < -0.2 and *p* < 0.05).
